# Testing Whether Established Risk Factors for Future Eating Disorder Onset Predict Future Overweight/Obesity Onset: A Prospective Study

**DOI:** 10.21203/rs.3.rs-7230160/v1

**Published:** 2025-08-06

**Authors:** Eric Stice, Yuko Yamamiya

**Affiliations:** Stanford University; Temple University, Japan Campus

**Keywords:** overweight, obesity, eating disorders, adolescent girls, adult women, risk factors, interactions

## Abstract

**Background/Objectives:**

The evidence that overweight and obesity often cooccur with eating disorders, overeating and binge eating increase risk for future eating disorder onset, and a prevention program that reduces overeating prevents future eating disorder onset suggests factors that increase risk for eating disorders may also increase risk for unhealthy weight gain. We test whether predictors of future eating disorder onset, which include both risk factors and prodromal symptoms, also predict future onset of overweight or obesity.

**Subjects/Methods:**

Data were collected from 1 952 adolescent girls and young women who completed annual assessments over a 3-year period. Among them, our final sample consisted of 1 669 participants (*Mean* age = 19.4, *SD* = 4.9) who met the inclusion criteria. Logistic regression models tested whether each established eating disorder risk factor predicted future onset of overweight or obesity. Classification tree analysis tested for interactions among the predictors.

**Results:**

Body dissatisfaction (OR = 1.43, 95% CI [1.23, 1.66], *p* < .001), negative affect (OR = 1.20, 95% CI [1.05, 1.37], *p* = .006), and feeling fat (OR = 1.37, 95% CI [1.19, 1.58], *p* < .001) increased risk for future onset of overweight/obesity and lower-than-expected body weight reduced risk (OR = 0.62, 95% CI [0.37, 0.83], *p* = .014), though only body dissatisfaction (OR = 1.25, 95% CI [1.04, 1.51], *p* = .017) and lower-than-expected body weight (OR = 0.65, 95% CI [0.38, 0.87], *p* = .026) showed unique predictive effects in a multivariate model. The classification tree model indicated that high body dissatisfaction showed the strongest predictive effect, and that elevated negative affect further amplified risk; results also revealed a distinct risk pathway characterized by low psychosocial impairment.

**Conclusions:**

Results identified several risk and protective factors for overweight/obesity onset, which may work together in a synergistic faction to increase risk for overweight/obesity.

## Introduction

Obesity is the leading cause of global mortality and morbidity, accounting for more than 4 million deaths each year, and reduces the lifespan by 7 years on average^1–2^, primarily due to heart disease, cerebral vascular disease, diabetes, and various cancers^1,3^. However, the most common treatment for obesity, behavioral weight loss interventions, rarely result in lasting weight loss^4^ and most obesity prevention programs do not prevent future obesity onset^5^. An improved understanding of the factors that increase risk for future unhealthy weight gain should guide the design of more effective obesity prevention programs and treatments.

Emerging data reveal that overweight and obesity often co-occur with eating disorders^6^. Prospective data reveal that the first behavioral eating disorder symptom to emerge when people develop various eating disorders, including anorexia nervosa (AN), bulimia nervosa (BN), binge eating disorder (BED), and purging disorder (PD), is unhealthy weight control behaviors^7^. In addition, overeating and binge eating predict future eating disorder onset^7–9^ and persistence of eating disorder symptoms^10^. Further, a meta-analytic review revealed that an intervention that promotes lifestyle changes to bring energy intake into balance with energy expenditure is one of only two strategies that has prevented future onset of eating disorders in multiple trials^11^. Collectively, these findings suggest that it would be useful to investigate whether risk factors that have been found to predict future onset of eating disorders also predict future onset of overweight and obesity to advance knowledge about risk factors for unhealthy weight gain and inform the design of more effective obesity prevention programs and treatments.

Prospective studies have established that pursuit of the thin beauty ideal, body dissatisfaction, self-objectification, dieting, negative affect, and social support deficits predicted future onset of any eating disorder^12–18^. Further, pursuit of the thin ideal, body dissatisfaction/weight concerns, dieting, weight suppression, negative affect, psychosocial impairment, and prodromal eating disorder symptoms, including binge eating, compensatory weight control behaviors, weight/shape overvaluation, fear of weight gain, feeling fat, and lower-than-expected body weight, predicted future onset of AN, BN, BED, and/or PD^7,19–22^.

Only a few of prospective studies have tested whether risk factors that have predicted future onset of eating disorders predict future onset of overweight or obesity. Past studies have found that dieting, depressive symptoms, compensatory weight control behaviors, and binge eating predicted future onset of overweight or obesity^23–25^. To address this research question, we analyzed data from a large sample of adolescent girls and young women who provided data on established risk factors for eating disorders at baseline and completed annual assessments over a 3-year follow-up period. We combined data from four large prospective randomized trials of eating disorder prevention programs to address this research aim. This sample allowed us to test whether eating disorder risk factors and prodromal symptoms assessed at baseline increase risk for future emergence of overweight or obesity over 3-year follow-up. We predicted future onset of overweight or obesity because these specific levels of excess adiposity have been found to increase risk for morbidity and mortality^26^. The prevention trials focused on biological women because they are at greater risk for eating disorders, which seemed acceptable given that biological women are also at elevated risk for overweight and obesity^1^.

## Methods

### Participants and Procedures

We combined data from one efficacy trial (Trial 1^27^ [completed prior to ClinicalTrials.gov]), two effectiveness trials (Trial 2^28^ [ClinicalTrials.gov identifier: NCT00663754]; Trial 3^29^ [ClinicalTrials.gov identifier: NCT01126918]), and one task-shifting implementation trial (Trial 4^30^ [ClinicalTrials.gov identifier: NCT01949649]) resulting in a sample of 1 952 participants who provided baseline data. In the combined sample, the average age of participants was 19.7 years (SD = 5.7) and 66% of participants were White, 10% Asian, 8% Hispanic, 5% Black, 2% Native Americans, 1% Pacific Islanders, and 8% multiracial. Parents of participants were well educated (71% were college graduates, of which 35% held advanced degrees). Data were collected in Texas, Oregon, and Pennsylvania, suggesting that results should be more generalizable than if the data were collected in only one region.

### Design of Randomized Prevention Trials

Mailings and fliers recruited cisgender females for trials evaluating body acceptance interventions at high schools (Trial 1 and 2) and colleges (Trial 1, 3, and 4). The sole inclusion criterion was that participants answer affirmatively when asked if they had body image concerns. Informed consent was obtained from participants (and parents for minors). Trial 1 participants were randomized to the *Body Project* eating disorder prevention program, *Healthy Weight* eating disorder prevention program, an expressive writing intervention, or assessment-only control condition. Trial 2 and 3 participants were randomized to the *Body Project* or educational brochure control condition. Trial 4 participants were randomized to clinician-led *Body Project* groups, peer-led *Body Project* groups, the Internet-delivered *eBody Project*, or an eating disorder education video control condition. Participants completed questionnaires and interviews at baseline and at 1-, 6-, 12-, 24-, and 36-month follow-up. Additional details can be found in Stice et al. (2021)^7^.

### Measures

#### Thin ideal internalization.

The 8-item Ideal-Body Stereotype Scale–Revised assessed pursuit of the thin ideal^10^. It has shown internal consistency (α = .91), 2-week test–retest reliability (*r* = .80), and predictive validity for future BN, BED, and PD onset (α = .69).^7^

#### Body dissatisfaction.

The 9-item Body Dissatisfaction Scale^31^ assessed dissatisfaction with various body parts. It has shown internal consistency (α = .94), 3-week test–retest reliability (*r* = .90), and predictive validity for future BN, BED, and PD onset (α = .84)^7^.

#### Dietary restraint.

The 10-item Dutch Restrained Eating Scale^32^ assessed the frequency of dieting behaviors. It has shown internal consistency (α = .95), 2-week test–retest reliability (*r* = .82), and predictive validity for future BN, BED, and PD onset (α = .92)^7,32^.

#### Negative affect.

Different measures of negative affect were used in the trials and were standardized to permit analyses in the combined sample. In Trials 1 and 4, negative affect was assessed with 20 items from the negative affect subscale from the Positive Affect and Negative Affect Scale-Revised (PANAS-X)^33^, which has shown internal consistency (α = .95), 3-week test-retest reliability (*r* = .78), and predictive validity for bulimic symptom onset^30^. In Trial 2, negative affect was assessed with the 20-item Center for Epidemiologic Studies-Depression Scale^34^, which has shown internal consistency (α = .74–.91), temporal reliability (2- to 8-week test-retest *r* = .51–.59), and convergent validity with clinician ratings of depressive symptoms (mean *r* = .88)^35^. In Trial 3, negative affect was assessed with the 21-item Beck Depression Inventory^36^, which has shown internal consistency (α = .73–.95), 1-week test-retest reliability (*r* = .93), and convergent validity with clinician ratings of depressive symptoms (mean *r* = .75)^10,36^. The PANAS-X negative affect subscale correlates with depressive symptom scales (mean *r* = .63)^33^, suggesting it is reasonable to average across these measures. This negative affect composite has shown predictive validity for future AN, BN, BED, and PD onset (α = .94)^7^.

#### Psychosocial impairment.

Impairment in psychosocial functioning in the family, peer group, romantic, and school or work domains was measured with 17 items from the Social Adjustment Scale-Self Report for Youth^37^. This scale has shown internal consistency (α = .77), 1-week test–retest reliability (*r* = .83), predictive validity for future AN, BN, BED, and PD onset (α = .74)^7^.

#### Prodromal eating disorder symptoms.

The Eating Disorder Diagnostic Interview (EDDI)^10^ assessed eating disorder symptoms over the past 3 months at baseline, which allowed us to test whether continuous variables reflecting the degree of each eating disorder symptom at baseline (prodromal symptoms) predicted future onset of overweight/obesity over 3-year follow-up. Regarding behavioral symptoms, we focused on frequency of binge eating, frequency of compensatory weight control behaviors (vomiting, laxative/diuretic use, fasting, and excessive exercise), and lower-than-expected body weight at baseline. Regarding cognitive symptoms, we focused on degree of overvaluation of weight and shape, fear of weight gain, and feeling fat in the past 3 months at baseline. To assess test-retest reliability, a randomly selected subsample of participants (*N* = 351) repeated the EDDI with the same assessor 1 week later. Test-retest reliability was κ = .94 for binge eating, κ = .86 for compensatory behaviors, κ = .80 for overvaluation of weight/shape, κ = .89 for fear of weight gain, and κ = .83 for feeling fat. To assess inter-rater reliability, a separate randomly selected subsample (*N* = 330) completed the EDDI with a second assessor within 1 to 3 days. Inter-rater reliability was κ = .51 for binge eating, κ = .77 for compensatory behaviors, κ = .89 for overvaluation of weight/shape, κ = .86 for fear of weight gain, and κ = .85 for feeling fat. Female assessors with a B.A./B.S., M.A., or Ph.D. in psychology attended 24 hours of training in which they were taught structured interview skills, reviewed diagnostic criteria for eating disorders, observed simulated interviews, and role-played interviews. Assessors were required to demonstrate an inter-rater agreement (κ > .80) with supervisors on 12 tape-recorded interviews prior to collecting data. Assessors completed annual refresher training to prevent diagnostic drift.

#### Overweight and Obesity.

The Body Mass Index (BMI; kg/m^2^)^38^ was used to reflect height-adjusted body mass. Height was measured to the nearest mm using portable stadiometers. Weight was assessed to the nearest 0.1 kg using digital scales with participants wearing light indoor clothing without shoes or coats. Height and weight were measured twice at each assessment and averaged. Age- and sex-adjusted BMI centiles were used to determine whether participants had a lower-than-expected BMI for their age and sex, which was examined as prodromal eating disorder symptom. Following convention^39^, overweight was defined as BMI between the 85th and 95th percentile and obese was defined as BMI greater than or equal to the 95th, based on the Centers for Disease Control and Prevention (CDC) centiles charts^40^. BMI has shown convergent validity (*r* = .80–.90) with measures of body fat such as dual energy x-ray absorptiometry^38^ and predictive validity for future AN onset^10^. Our primary outcome variable was onset of overweight or obesity among those with a healthy BMI at baseline or a transition from overweight to obese during the 3-year follow-up.

### Statistical Methods

We first estimated univariate models to test whether any risk factors and/or prodromal symptoms at baseline predicted a future onset of overweight or obesity. We next entered the risk factors that showed univariate effects into a multivariate model to determine the unique predictive effect of each risk factor, controlling for the other risk factors that showed univariate effects. Additionally, we performed a classification tree analysis using all risk factors and prodromal symptoms to predict future onset of overweight/obesity, which is an exploratory analytic technique that can detect interactions between the risk factors in predicting onset of a dichotomous outcome. We selected a classification tree analyses because it can both identify the most potent signal predictor and detect interactions between predictors. All major analyses were conducted in R^41^.

### Missingness

Data were missing from 5%, 10%, 7%, 10%, and 17% at 1-month, 6-month, 1-year, 2-year, and 3-year follow-up, respectively.

## Results

### Preliminary Analyses

**Figure 1.**
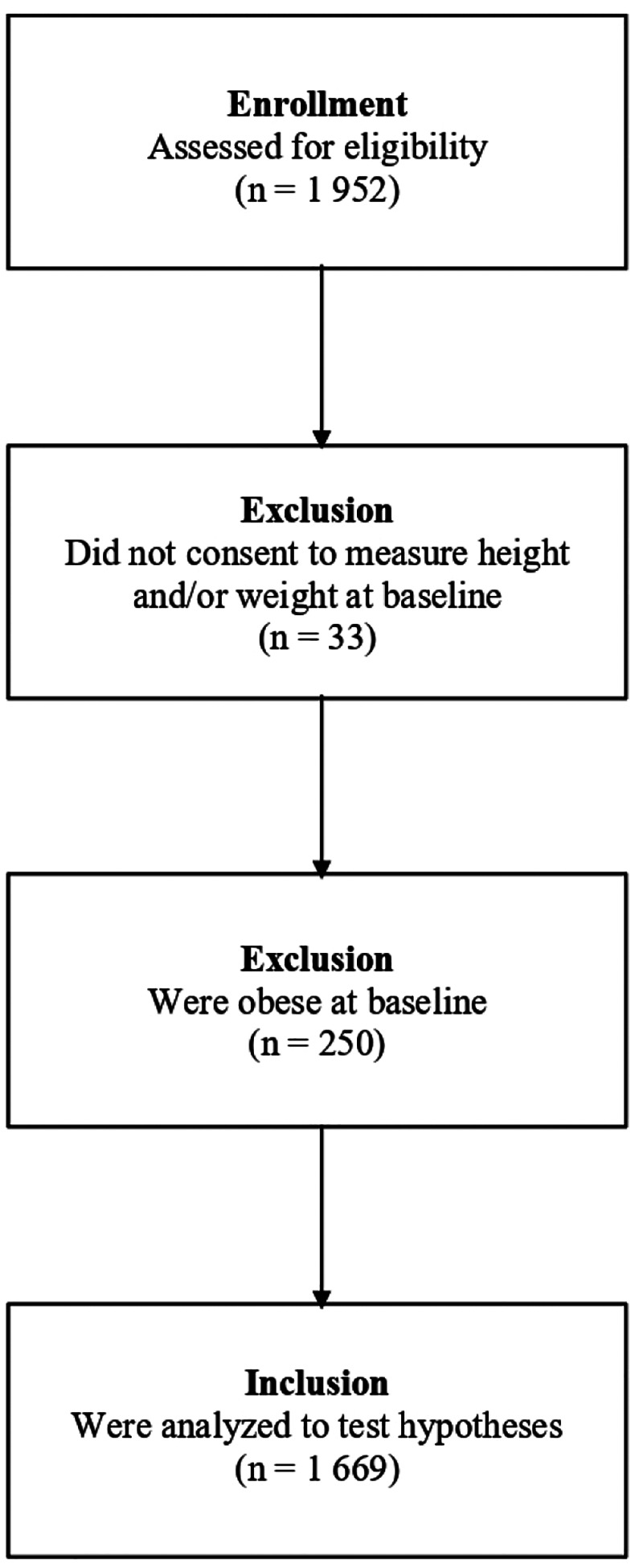
Participant Flow Diagram.

Regarding participants’ baseline weight status, we excluded 33 individuals who did not consent to be weighed at baseline because we could not determine whether they showed onset of overweight or obesity over follow-up. We also excluded 250 participants who were already classified as obese at baseline. The final sample consisted of 1 669 participants who self-identified as adolescent girls/women, whose mean age at baseline was 19.4 years (*SD* = 4.9), and their mean baseline BMI was 23.0 (*SD* = 3.0). See Fig. 1 for the participant flowchart.

### Univariate Predictors of Future Overweight/Obesity Onset

To examine whether the eating disorder risk factors and/or prodromal symptoms significantly predicted future onset of overweight/obesity, we first estimated univariate logistic regression models. Significant univariate predictors were body dissatisfaction (OR = 1.43, 95% CI [1.23, 1.66], *p* < .001) with a small-to-medium effect size (SRD = 0.20), which translates to an absolute risk of 20% of developing obesity over three years compared to the baseline risk of 15% (i.e., 15% of the sample developed obesity over follow-up), negative affect (OR = 1.20, 95% CI [1.05, 1.37], *p* = .006) with a small effect size (SRD = 0.14), which translates to an absolute risk of 18% of developing obesity over follow-up compared to the baseline risk, feeling fat (OR = 1.37, 95% CI [1.19, 1.58], *p* < .001) with a small-to-medium effect size (SRD = 0.18), which translates to an absolute risk of 20% of developing obesity over follow-up compared to the baseline risk, and lower-than-expected BMI (OR = 0.62, 95% CI [0.37, 0.83], *p* = .014) with a small effect size (SRD = −0.04), which translates to an absolute risk of 10% of developing obesity over follow-up compared to the baseline risk (Table 1). When the Benjamini-Hochberg correction for multiple testing was applied, all the *p*-values remained significant.

### Multivariate Predictors of Future Overweight/Obesity Onset

To examine the unique effects of these risk factors after accounting for the effects of the other variables in the model, we estimated a multivariate logistic regression model that included body dissatisfaction, negative affect, feeling fat, and lower-than-expected BMI (Table 2). Two variables remained significant: body dissatisfaction (OR = 1.25, 95% CI [1.04, 1.51], *p* = .017) and lower-than-expected BMI (OR = 0.65, 95% CI [0.38, 0.87], *p* = .026) with small effect sizes (SRDs = .04 and .02, respectively), reflecting small unique effects. The predicted probability of developing obesity increased from 11–17% for participants with 1 *SD* above vs. 1 *SD* below the mean on body dissatisfaction. In contrast, a 1 *SD* increase in lower-than-expected BMI was associated with an absolute risk reduction of 11% for overweight/obesity onset, decreasing from 20–9%. Negative affect and feeling fat did not show significant unique effects. When the Benjamini-Hochberg correction for multiple testing was applied, both of the significant *p*-values became marginally significant (*ps* = .052).

### Multivariate Interactions between Predictors of Future Overweight/Obesity Onset

**Figure 2.**
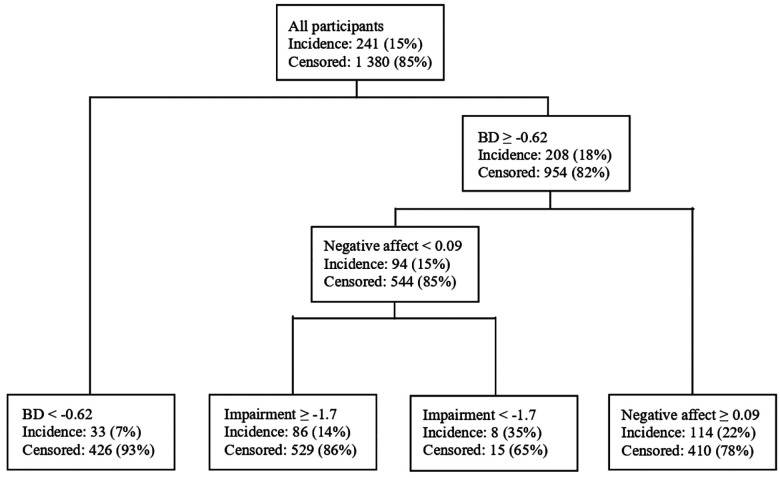
Classification Tree Identifying Predictors of Abnormal Weight Transition *Note*. Standardized scores are used.

A classification tree analysis was conducted to identify the key predictors of overweight/obesity onset, using the default class priors (i.e., no specified priors), a complexity parameter of 0.001, a minimum split size of 20, and a minimum terminal node size of 10. The analysis yielded an overall accuracy of 0.67, sensitivity of 0.50, and specificity of 0.70, with an area under the curve (AUC) of 0.63 based on a decision threshold of 0.15. The first split was based on body dissatisfaction, indicating it was the most potent single predictor. Participants with high body dissatisfaction had an absolute risk of 18%, compared to the overall baseline risk of obesity onset was 15%. The second split was based on negative affect, which communicated that for participants with higher body dissatisfaction, negative affect was the next most potent predictor. Participants with both high body dissatisfaction and high negative affect had an absolute risk of 22%. In contrast, among participants with relatively lower negative affect, lower psychosocial impairment increased risk future onset of overweight/obesity (Fig. 2). This subgroup showed the highest observed absolute risk, with 35% of participants meeting the outcome. Two terminal nodes, which together accounted for less than 2% of the total sample, were not reported here or in the figure due to their small size and limited interpretability. Thus, results suggests that negative affect amplifies the relation between body dissatisfaction and risk for overweight/obesity onset, and provided evidence that distinct risk pathways involving low psychosocial impairment. These results indicated that the model was fairly effective in detecting true positives, although some false negatives may occur. However, this may be due to the imbalanced dataset, in which the majority (90%) of the entire participants did not show obesity onset over follow-up.

## Discussion

To the best of our knowledge, this is the first study to provide evidence that elevated body dissatisfaction, negative affect, and feeling fat increased risk for future overweight/obesity onset. The evidence that negative affect increased risk for overweight/obesity onset appears to converge with the finding that depressive symptoms increased risk for future onset of overweight/obesity, observed in a prior study^24^. The evidence that lower-than-expected body weight served as a protective factor against future onset of overweight/obesity is also a novel finding that has not been reported previously. The effect sizes were small to medium in magnitude. We did not replicate evidence that binge eating, dieting, and compensatory behaviors predicted future onset of overweight/obesity in this sample, which was observed in previous prospective studies^23–25^.

Regarding unique predictive effects, among the four risk factors that showed significant univariate effects, body dissatisfaction and lower-than-expected BMI showed unique and independent relations to onset of overweight/obesity after controlling for the predictive effects of the other variables. The significant univariate effect for feeling fat likely became non-significant because, as shown in Table 3, it was colinear with body dissatisfaction (*r* = .56). Similarly, the predictive effect of negative affect may have become non-significant because it was also colinear with body dissatisfaction (*r* = .39).

The classification tree model suggested that body dissatisfaction was the most potent single risk factor that predicted future onset of overweight/obesity in this sample. Participants with higher body dissatisfaction showed more than a twofold increase in the incidence for overweight/obesity onset compared to those with lower body dissatisfaction (18% vs. 7%). This finding aligns with a study of women with obesity that demonstrated a significant association between body dissatisfaction and binge eating behavior, even after controlling for BMI^42^. However, when body dissatisfaction was reduced through an intervention, binge eating behaviors also decreased, even after controlling for weight loss. This suggests that it might be useful to test if interventions that have been found to significantly reduce body dissatisfaction^27–30,43^ are effective in preventing onset of overweight/obesity. The classification tree model also provided evidence that among participants with high body dissatisfaction, negative affect emerged as the next most potent predictor. That is, the risk is amplified further by elevated negative affect among those with high body dissatisfaction compared to those with low negative affect (22% vs. 15%). This sequential link accords with the dual pathway model of eating pathology, which postulates that body dissatisfaction contributes to negative affect, which in turn leads to unhealthy eating behaviors^44^. Binge eating may be used to address negative affect, consistent with evidence that it mediates the predictive effect of depressive symptoms on future obesity^45^. These results suggest interventions that have been found to reduce negative affect^27–30,46^ might prove useful in preventing obesity onset. In contrast, among participants with relatively lower negative affect, psychosocial impairment emerged as an additional pathway to overweight/obesity onset. That is, among those with high body dissatisfaction and lower negative affect, participants who reported lower impairment in their psychosocial functioning had more than a two-fold higher risk of overweight/obesity onset (35% vs. 14%). One possibility is that the model is overfitting due to the small number of observations in the node (n = 23) and/or the previously mentioned data imbalance. Psychosocial impairment and interpersonal problems have been found to be associated with pathological eating behaviors (e.g., binge eating, purging)^7,9^, so further studies are needed to explore how psychosocial functioning is related to obesity onset with a larger sample. In sum, it is possible that it is easier to reduce these risk factors than to directly target reductions in caloric intake, which might be a more efficient method of preventing unhealthy weight gain. Indeed, these prevention program may also prove effective in reducing future onset of eating disorders.

### Limitations

It is important to consider study limitations. First, all participants endorsed a known eating disorder risk factor—body image concerns—for study inclusion, creating a higher risk sample. Thus, the predictive effects identified in this report may not generalize to adolescent girls/young women who are satisfied with their bodies. Second, a portion of participants in all four trials (about 50%) were offered an eating disorder prevention intervention after providing baseline data on risk factors, which may have affected risk for onset of overweight or obesity over follow-up. Third, this sample included only biological women, so findings may not generalize to biological men. Fourth, it would have been ideal to have followed the participants for a longer follow-up period because it would have increased sensitivity. Fifth, we combined data from four samples, which may have introduced inconsistencies in methodology or population characteristics. Finally, classification tree analysis is an exploratory hypothesis generating analytic approach, so the findings should be interpreted with that in mind.

## Conclusions

Obesity is associated with various medical issues, including heart disease, cerebral vascular disease, diabetes, and numerous types of cancer^1,3^, and is one of the leading causes of mortality and morbidity^1,2^. Hence, it is important to advance knowledge of psychosocial factors that increase risk of future unhealthy weight gain. Our findings suggest that body dissatisfaction, negative affect, feeling fat, and psychosocial impairment increase risk for unhealthy weight gain, whereas lower-than-expected BMI served as a protective factor. Results further indicated that body dissatisfaction showed the most potent predictive effects, followed by negative affect. This suggests that the prevention programs that have been shown to reduce body dissatisfaction and negative affect may prove most useful in preventing future unhealthy weight gain.

## Figures and Tables

**Table 1:** Univariate Logistic Regression Models Where Abnormal Weight Gain was Regressed onto Risk Factors and Prodromal Symptoms

Predictors	LOR	OR	SE	Z	*p*	OR 95% CI		SRD
Risk factors								
Thin-ideal internalization	0.08	1.08	0.07	1.13	.258	0.94	1.25	0.04
Body dissatisfaction	0.36	1.43	0.08	4.70	**< .001**	1.23	1.66	0.20
Negative affect	0.18	1.20	0.07	2.76	**.006**	1.05	1.37	0.14
Dietary restraint	0.10	1.11	0.07	1.50	.134	0.97	1.27	0.06
Psychosocial impairment	0.08	1.08	0.07	1.13	.257	0.94	1.24	0.05
Prodromal symptoms								
Binge eating	0.01	1.01	0.07	0.15	.878	0.87	1.15	0.05
Compensatory behaviors	−0.03	0.97	0.07	−0.41	.684	0.83	1.12	0.02
Weight/shape overvaluation	0.10	1.10	0.07	1.34	.181	0.96	1.27	0.04
Fear of weight gain	0.06	1.06	0.07	0.86	.392	0.92	1.22	0.05
Feeling fat	0.31	1.37	0.07	4.40	**< .001**	1.19	1.58	0.18
Lower-than-expected BMI	−0.48	0.62	0.19	−2.46	**.014**	0.37	0.83	−0.04

*Note*. LOR = log odds ratio; OR = odds ratio; SRD = success rate difference. SRDs of 0.11, 0.28, and 0.43 correspond to small, medium and large effects, respectively.

**Table 2:** Multivariate Logistic Regression Models Where Abnormal Weight Transition was Regressed onto Risk Factors and Prodromal Symptoms

Predictors	LOR	OR	SE	Z	*p*	OR 95% CI		SRD
Risk factors								
Body dissatisfaction	0.22	1.25	0.09	2.40	**0.017**	1.04	1.51	0.04
Negative affect	0.06	1.06	0.07	0.80	0.425	0.92	1.23	0.01
Prodromal symptoms								
Feeling fat	0.13	1.14	0.09	1.48	0.139	0.96	1.36	< 0.01
Lower-than-expected BMI	−0.43	0.65	0.19	−2.23	**0.026**	0.38	0.87	0.02

*Note*. LOR = log odds ratio; OR = odds ratio. SRDs of 0.11, 0.28, and 0.43 correspond to small, medium and large effects, respectively.

**Table 3:** Correlation Matrix of 11 Independent Variables

Variables	2	3	4	5	6	7	8	9	10	11
1. Thin-ideal internalization	**.280** **	**.214** **	**.312** **	**.052** *	**.058** **	**.150** *	**.262** **	**.175** **	**.271** **	−.091**
2. Body dissatisfaction	-	**.388** **	**.399** **	**.203** **	**.169** **	**.263** **	**.367** **	**.352** **	**.560** **	−.124**
3. Negative affect		-	**.297** **	**.524** **	**.213** **	**.269** **	**.361** **	**.315** **	**.359** **	.012
4. Dietary restraint			-	**.088** **	**.146** **	**.401** **	**.448** **	**.431** **	**.463** **	− .209**
5. Psychosocial impairment				-	**.192** **	**.188** **	**.149** **	**.215** **	**.161** **	.038
6. Binge eating					-	**.160** **	**.154** **	**.170** **	**.199** **	−.006
7. Compensatory behaviors						-	**.338** **	**.349** **	**.319** **	− .068**
8. Weight/shape overvaluation							-	**.378** **	**.465** **	−.065**
9. Fear of weight gain								-	**.543** **	− .097**
10. Feeling fat									-	−.160**
11. Lower-than-expected BMI										

*Note*.

* *p* < .05,

** *p* < .01.

## Data Availability

Data described in the manuscript, the codebook, and analytic code will be made available upon reasonable request for academic use.
